# Characterization of Metabolite Profile in *Phyllanthus niruri* and Correlation with Bioactivity Elucidated by Nuclear Magnetic Resonance Based Metabolomics

**DOI:** 10.3390/molecules22060902

**Published:** 2017-05-30

**Authors:** Ahmed Mediani, Faridah Abas, M. Maulidiani, Alfi Khatib, Chin Ping Tan, Intan Safinar Ismail, Khozirah Shaari, Amin Ismail

**Affiliations:** 1 Department of Food Science, Faculty of Food Science and Technology, Universiti Putra Malaysia, 43400 Serdang, Selangor, Malaysia; medianiahmed47@gmail.com; 2 Laboratory of Natural Products, Institute of Bioscience, Universiti Putra Malaysia, 43400 Serdang, Selangor, Malaysia; dieni_maulydia@yahoo.com (M.M.); alfikhatib1971@gmail.com (A.K.); safinar@upm.edu.my (I.S.I.); khozirah@upm.edu.my (K.S.); 3 Department of Pharmaceutical Chemistry, Faculty of Pharmacy, International Islamic University Malaysia, 25200 Kuantan, Pahang, Malaysia; 4 Department of Food Technology, Faculty of Food Science and Technology, Universiti Putra Malaysia, 43400 Serdang, Selangor, Malaysia; tancp@upm.edu.my; 5 Department of Chemistry, Faculty of Science, Universiti Putra Malaysia, 43400 Serdang, Selangor, Malaysia; 6 Department of Nutrition and Dietetics, Faculty of Medicine and Health Sciences, Universiti Putra Malaysia, 43400 Serdang, Selangor, Malaysia; aminis@upm.edu.my

**Keywords:** *Phyllanthus niruri*, NMR spectroscopy, α-glucosidase inhibitory activity, amino acids, metabolomics

## Abstract

*Phyllanthus niruri* is an important medicinal plant. To standardize the extract and guarantee its maximum benefit, processing methods optimization ought to be amenable and beneficial. Herein, three dried *P. niruri* samples, air (AD), freeze (FD) and oven (OD), extracted with various ethanol to water ratios (0%, 50%, 70%, 80% and 100%) were evaluated for their metabolite changes using proton nuclear magnetic resonance (^1^H-NMR)-based metabolomics approach. The amino acids analysis showed that FD *P. niruri* exhibited higher content of most amino acids compared to the other dried samples. Based on principal component analysis (PCA), the FD *P. niruri* extracted with 80% ethanol contained higher amounts of hypophyllanthin and phenolic compounds based on the loading plot. The partial least-square (PLS) results showed that the phytochemicals, including hypophyllanthin, catechin, epicatechin, rutin, quercetin and chlorogenic, caffeic, malic and gallic acids were correlated with antioxidant and α-glucosidase inhibitory activities, which were higher in the FD material extracted with 80% ethanol. This report optimized the effect of drying and ethanol ratios and these findings demonstrate that NMR-based metabolomics was an applicable approach. The FD *P. niruri* extracted with 80% ethanol can be used as afunctional food ingredient for nutraceutical or in medicinal preparation.

## 1. Introduction

The economic viability and pharmacological potential of plant-derived drugs are prominent and current research focuses on tropical countries due to their large varieties of medicinal herbs. Part of the attraction to these herbs is their potential for natural protection against chronic diseases because of their high levels of antioxidants to scavenge free radicals, which are the leading cause of most maladies [1,2,3,4]. Plants and their products have also received much attention from the food industry because of their pleasant flavor and natural bioactivity [5]. The limitation of resources of most people and the unfeasibility of modern drugs are also essential factors when focusing on the plant as a replacement cure to several chronic diseases. Conventional remedies were linked to recent studies on plant material to establish standardization for plant-derived drugs.

*Phyllanthus niruri* (Euphorbiaceae) is an annual herbal plant that is spread worldwide, especially in tropical countries. In Malaysian communities, it is a well-known plant that is locally named ‘dukunganak’. It is also known as ‘paraparai mi’ in Paraguay, ‘quebra pedra’ in Brazil, ‘chancapiedra’ in Spanish and ‘punarnava’ in India [6,7]. It has traditionally been used as a pain reliever, appetite stimulator, antihelmintic, diuretic and has an emmenagogue effect [8,9]. Numerous metabolite classes of great medicinal significance have been previously identified in *P. niruri*, including flavonoids, alkaloids, triterpenes, tannins, lignans, polyphenols and sterols [6]. In terms of the richness of these efficient medicinal metabolites, various therapeutic activities are associated with *P. niruri*, including antiviral, antimicrobial, antihepatic, antitumor and antidiabetic activities [9]. The flavonoids in this plant include rutin; gallocatechin; quercetin 3-*O*-glucopyranoside; quercetin; kaempferol 3-*O*-d-glucopyranoside and kaempferol. Ellagitannins were also identified in *P. niruri*; such as geraniin; furosin; amariin; amariinic acid; geraniinic acid B; repandusinic acid A; amarulone; corilagin; elaeocarpusin; phyllanthusiin A, B, C and D; isocorilagin; and melatonin [9,10]. The presence of these compounds in *P. niruri* makes it of great interest to researchers who are interested in determining its health benefits and studying the effect of processing on these metabolites.

Metabolomics is a comprehensive tool targeting the evaluation of the variation in the metabolites of organisms under different conditions [11,12]. Metabolomics also provides the immediate detection of a wide range of metabolites, providing a simultaneous picture about the organism metabolome. Metabolomic approaches can be used to study any biological system. Naturally, plants can be highlighted as the subject of study [12,13,14]. Processing techniques could change the significance and level of metabolites. The extraction with various solvents is also an aspect of the variance in the benefit and amount of metabolites [6,15,16]. Metabolomics is based on several techniques to measure variation, and nuclear magnetic resonance (NMR) is one of the leading analytical techniques for monitoring and discriminating these differences in several scientific areas [17,18,19,20]. The NMR and mass spectrometry (MS) have proved to be suitable for examining the metabolites’ composition and changes as well as simultaneously quantifying a range of metabolites, altering or contributing to that of automated analysis. Both tools have their own advantages and disadvantages; MS is more sensitive than NMR. However, NMR can support the identification of a larger group of compounds than MS [12,18]. The NMR spectroscopy can provide an uncomplicated structural analysis obtained from the signals and coupling constants of primary and secondary metabolites present in organisms, including crude extracts [18]. In addition, it offers valuable information about the quality and quantity of metabolites due to the intensity of their signals relative to molar concentration [18]. Therefore, NMR analysis considers the number of identified metabolites and not the signals assigned. This makes NMR an excellent choice for the fingerprinting and discrimination of medicinal plants.

The recent widespread implementation of NMR as a tool for plant metabolomics is due to its significance and potential ability to detect various metabolite groups, such as amino acids, carbohydrates, phenolics, flavonoids and lignans [18]. However, published data on most of the metabolite variation in processed *P. niruri* are rare. Herein, NMR was applied to identify possible and candidate metabolites that might be changed for different dried *P. niruri* extracts. Therefore, the objective of this study was to analyze the metabolic differences among freeze-, air-, and oven-dried *P. niruri* samples extracted with different ethanol/water ratios (0%, 50%, 70%, 80%, 100%) by applying a ^1^H-NMR-based metabolomics approach. The correlation between the variation among processed samples and their antioxidant and α-glucosidase inhibitory activities was also investigated via partial least square (PLS) analysis. Research on the *P. niruri* metabolome warrants further investigation, although several researchers have previously detailed the medicinal effects of *P. niruri*.

## 2. Results and Discussion

### 2.1. Amino Acid Profile of the Dried Phyllanthus niruri

Amino acids are an important component from a nutritional point of view, especially essential amino acids, which the human body cannot synthesize them and that must be supplied from the diet. The freeze-dried sample had the highest amino acids content compared to other dried samples. Lysine, phenylalanine and leucine were the dominant essential amino acids in all dried samples and freeze-dried sample had the highest values with 14.10 ± 0.32, 4.01 ± 0.42 and 3.49 ± 0.08 mg/g, respectively (Table 1). This finding is correlated with the protein content because the freeze-dried sample had the highest value. Air- and oven-dried samples displayed lower amino acids content as compared to the freeze-dried one, which could be due to the effect of the associated conditions and mechanism involved. This result was contradicted with previous findings which showed that oven drying increases the content of amino acids better than freeze drying as compared to the raw material. Not only amino acids are important in terms of nutrition function, but they also act as precursors of phenolic compounds, such as cinnamic acid, *p*-coumaric acid, caffeic acid, ferulic acid, gallic acid, chlorogenic acid and protocatechuic acid, which play a role in bioactivity. These components are mostly produced from tyrosine and phenylalanine, which are essential amino acids [20,21].

### 2.2. ^1^H Nuclear Magnetic Resonance Spectra of the Samples and Metabolites Assignment

The 2D NMR (*J*-resolved and HMBC) spectra can be used to solve the problem of metabolic networks and the integration of NMR based metabolomics data. The *J*-resolved spectra facilitated the assignment of metabolites and provided some useful information regarding coupling constant and signal splitting of overlapped signals that confirmed the identification of assigned signals by 1D-NMR (Supplementary Figure S1). The identification of metabolites was also achieved by comparing them with the NMR signals of reference compounds under the same parameters, literature data, and Chenomx library [12,18]. Table 2 shows the identified compounds from *P. niruri* extracts and their characteristic signals. A total of 24 metabolites were identified, including primary and secondary metabolites (Supplementary Figure S2).

There was quantitative and qualitative variation for most metabolites. Numerous signals revealed in the aromatic and carbohydrate regions showed noticeable differences in the sample spectra. There was also clear variability in these regions, which might contribute to the variation among samples regarding chemical composition and bioactivity. Thus, these regions were given high priority for evaluating the variability among studied samples. In the carbohydrate regions, most assigned peaks were observed as overlapped signals, which were supported by several NMR studies [12,14]. The significant variability in the intensity and existence of peaks was clearly noted in the aromatic region of spectra; freeze-dried samples extracted with 70% and 80% ethanol showed most of these characteristics.

By visually inspecting the ^1^H-NMR spectra of all samples, several metabolite classes can be recognized. There was visible variation among the ^1^H-NMR spectra of the samples dried with three drying methods and extracted with different ethanol ratios, especially in aliphatic (δ 0.5–3.0) and aromatic (δ 5.5–9.0) regions. The metabolite identification and pattern recognition of *P. niruri* samples were quite complex in the carbohydrate region (δ 3.0–5.0) due to the overlapping signals of metabolites. The domination of some signals ascribed to quinic acid, glycoside and flavonoids in the aromatic region also varied among the extracts. To confirm the identified metabolites, especially in the aromatic region, *J*-resolved spectra showed these characteristic signals. For the flavonoid or glycoside metabolites (quercetin, catechin, rutin and kaempferol), their signals were identified and confirmed by the 2D NMR spectra.

### 2.3. Discrimination of the Dried Samples Extracted with Different Ethanol Ratios by Principal Component Analysis

**Figure 1:**
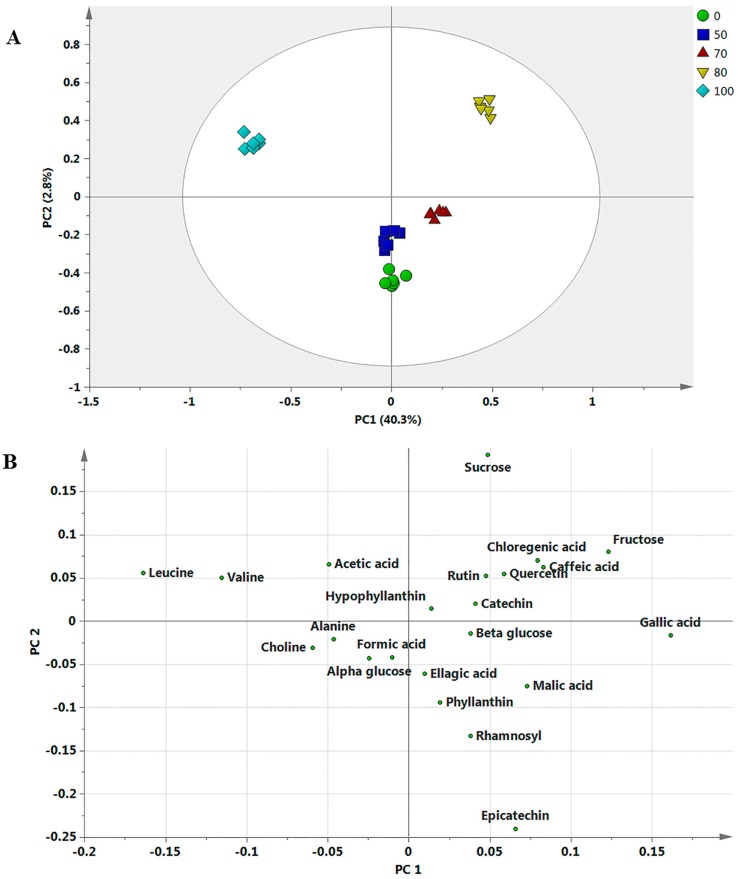
The principal component analysis (PCA) score (**A**) and loading (**B**) plots (PC1 vs. PC2) of the discrimination of *P. niruri* extracts with various ethanol ratios (0%, 50%, 70%, 80%, 100%); The numbers refer to the ethanol/water ratio; 100:100%, 80:80%, 70:70%, and 50:50%.

It is impossible to assess the differentiation among samples with a large dataset without applying the statistical analysis that reduces these data to manageable numbers. Thus, MVDA were further utilized to evaluate these variations in the constituent and level of metabolites of dried *P. niruri* samples extracted with different ethanol ratios. To obtain more detailed metabolic variations among *P. niruri* extracts, PCA was applied to the datasets. The PCA was applied to comprehend the clustering features of the samples and identify the metabolites that contributed to the variation. In the present study, PCA was carried out on the NMR data from the three dried *P. niruri* samples that were extracted with water and ethanol at different ratios (50%, 70%, 80% and 100%). To facilitate the comparison of dried samples and extracts with different solvent ratios, 80% EtOH solvent and FD methods were chosen as typical parameters according to previously reported bioactivity assays [16]. There were clusters separated by PCA without any moderate outliers. The PC1 indicated the highest variation, followed by PC2. The first two PCs exhibited a total of 70.1%, accounting for a variance of 40.3% by PC1 and 29.8% by PC2. The score plot of all samples revealed that three clusters were clearly identified (Figure 1). By comparing the solvents, 80% ethanol extracts of *P. niruri* were clearly discriminated from other extracts. The loading score plot showed the metabolites that contribute to the separation of samples. The 70% and 80% ethanol extracts of *P. niruri* had high levels of hypophyllanthin, catechin, epicatechin, rutin, quercetin and chlorogenic, ellagic, caffeic, malic and gallic acids compared to the others. The 100% ethanol extracts of *P. niruri* contained high amino acids content, which were responsible for its separation from other extracts.

**Figure 2:**
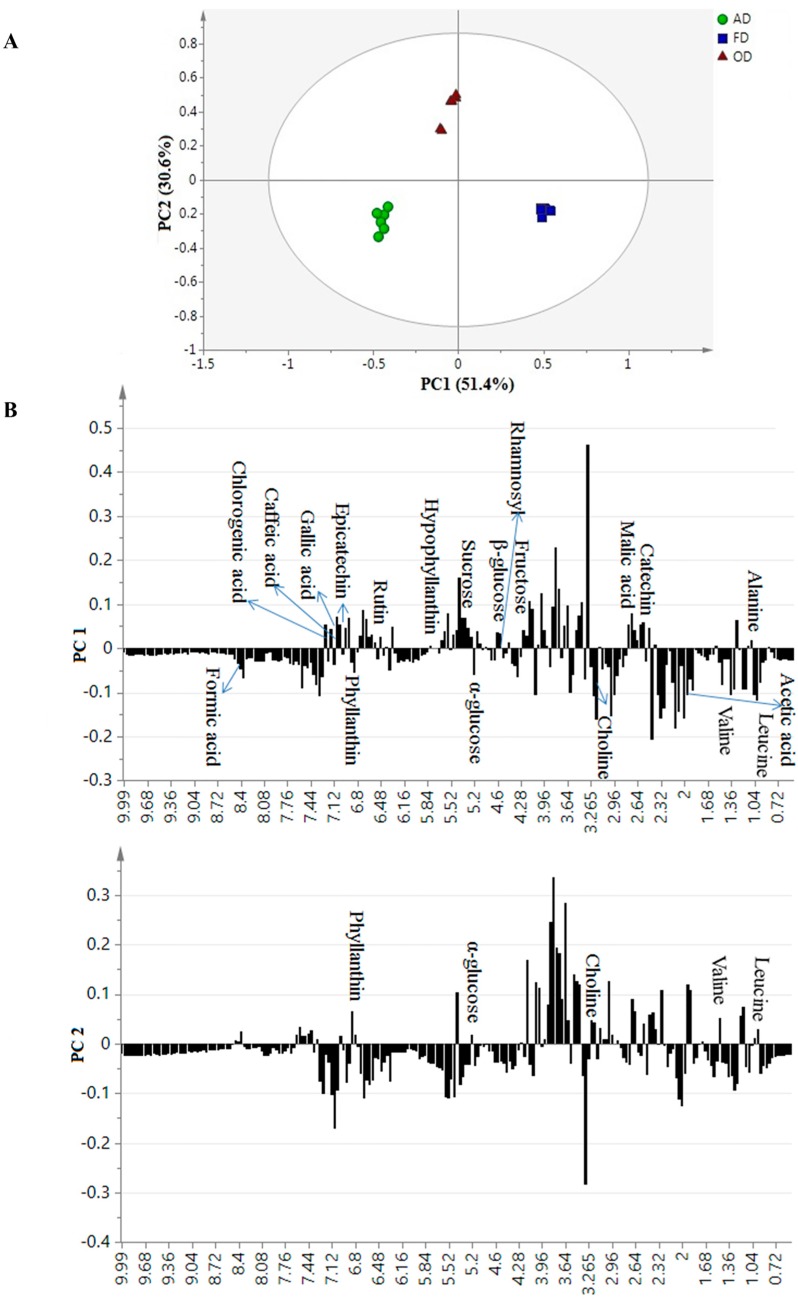
The PCA score plot (PC1 vs. PC2, (**A**)) and PC1 and PC2 loading column plots (**B**) of the discrimination among freeze drying (FD), oven drying (OD) and air drying (AD) of *P. niruri* extracts.

Figure 2 shows the PCA score and loading column plots. From the score plot, as a function of drying methods, three remarkable clusters were clearly observed (Figure 2). The first two PCs described a variation of 82.0%, where the major variance among samples was exhibited by PC1 with 51.4%, followed by PC2 with 30.6%. The score plot showed three clear clusters representing the OD, AD and FD samples. The FD samples were discriminated from AD and OD by PC1, where OD was separated from others by PC2. The FD *P. niruri* samples were completely separated from the others. The highest tested activity and phytochemical constituents explained the separation of the FD extracts from the others. Several researchers suggested that FD is the preferred drying method for retaining bioactive compounds [7,22]. Another study recommended that FD and AD are advantageous in preserving plant efficiency and bioactivity [13].

**Figure 3:**
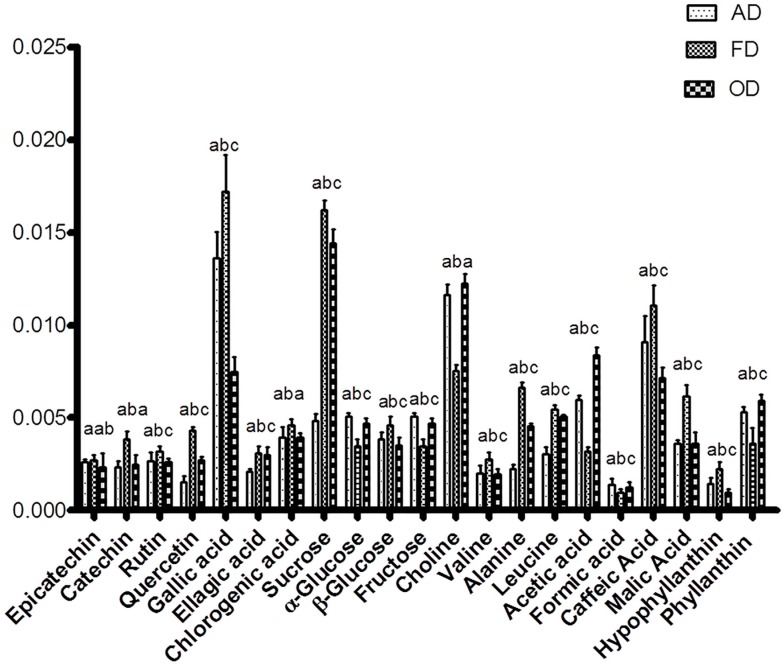
Relative quantification of identified metabolites of different dried *P. Niruri* based on the mean peak area of the ^1^H-NMR signals. The characteristic binned signals of epicatechin at δ 7.00 (s), quercetin at δ 7.36 (d), chlorogenic acid at δ 7.20 (s), catechin at δ 2.56 (dd), rutin at δ 6.52 (d), gallic acid at δ 7.08 (s), ellagic acid at δ 7.48 (s), formic acid at δ 8.48 (s), acetic acid at δ 1.92 (s), choline at δ 3.24 (s), α-glucose at δ 5.20 (d), β-glucose at δ 4.60 (d), sucrose at δ 5.40 (d), fructose at δ 4.18 (d), valine at δ 1.52 (d), alanine at δ 1.48 (d), leucine at δ 0.96 (d), phyllanthin at δ 6.84 (d), hypophyllanthin at δ 5.62 (d), malic acid atδ 2.72 (d) and caffeic acid at δ 7.12 (d). ^a,b,c^ Different letter above every columns indicates that the results showed statistically significant (*p* < 0.05; n = 6) differences.

For the OD sample, based on the present study, thermal treatment might cause degradation in most of the metabolites, especially phenolics, which are heat sensitive [22]. The phytochemical constituents, which contributed to the separation among the dried *P. niruri* materials, are illustrated in loading column plots (Figure 2). Most of the phytochemical constituents were located in the same quadrant as the freeze-dried materials, which confirmed all previous results. By combining the score and loading plots, most of the substances were highly concentrated in the FD samples. The FD and the 80% ethanol extracts were separated because of their high levels of hypophyllanthin, catechin, epicatechin, rutin, quercetin and chlorogenic, ellagic, caffeic, malic and gallic acids. Among the identified amino acids, only alanine was higher in FD samples, as previously shown by HPLC. By comparing the results obtained by HPLC with NMR, alanine, leucine and valine were detected by both tools. Due to the use of different solvents, the amino acids profiles by HPLC and NMR are different. These metabolites were also quantified relatively using NMR, and the concentration of most of them was significantly (*p* < 0.05) higher in FD samples (Figure 3). The advantage of the FD method compared to other drying methods was expected due to previous reports [7,23]. The mechanism associated with FD is based on dehydration by sublimation of the frozen product. Degradation by enzymatic and microbial processes is not preferred since low temperature is used during water removal [22]. The observed low concentration associated with AD in comparison to FD might be due to the action of enzymes, such as polyphenol oxidases. The enzyme activity does not effectively stop during AD, and water removal is slow, allowing microbial colonies to continue to thrive in the biomass and transform the metabolome of the plant material [23]. Similarly, the larger reduction of TPC in OD extract could be due to the effect of heat on the metabolites. The variation of these metabolites among the dried *P. niruri* extracts might affect their bioactivities.

### 2.4. Correlation between Bioactivities and Metabolite Changes among the Samples

**Figure 4:**
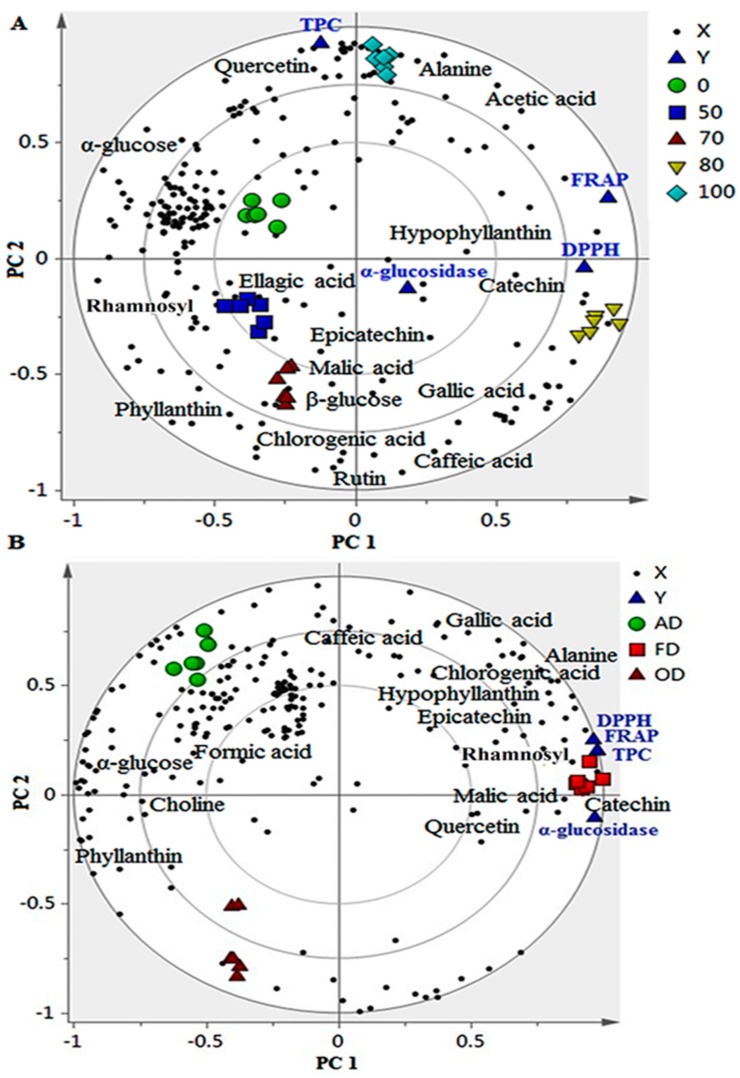
The biplot obtained from partial least square (PLS) describing the correlation among the phytochemical constituents of different ethanol extracts (**A**) of various dried (**B**) *P. niruri* total phenolic content (TPC), ferric ion reducing antioxidant power (FRAP), 2,2-diphenyl-1-picrylhydrazyl radical scavenging (DPPH), and α-glucosidase inhibitory activities. The numbers refer to the ethanol/water ratio; 100:100%, 80:80%, 70:70%, and 50:50%. AD—Air drying; OD—Oven drying; FD—Freeze drying.

The biological activity of plant samples is linked to several types of compounds. Phenolic compounds showed a prominent contribution to numerous bioactivities, including antioxidant and α-glucosidase activities [9]. Free radicals are the leading cause of most maladies, and phenolic compounds can scavenge these radicals to protect the biological system against oxidative stress. In this study, the applied in vitro assays were reported previously [16], including antioxidant activity analyzed by ferric reducing antioxidant potential (FRAP) and diphenylpicrylhydrazyl (DPPH), as well as α-glucosidase inhibitory activity and total phenolic content (TPC). The PLS model was implemented to show the correlation between these activities and the chemical constituents of the samples. This model is a supervised MVDA method used to obtain a maximal variation among datasets based on *X* variables and correlates them with *Y* variables. PLS was applied as a validated model to perform a degree of overfit on the dependent and independent variables [20]. The prediction performance of the PLS model is also a prominent advantage. The evaluation of variables influencing the bioactivities was evaluated by giving them the same chance by weightage data with the standard deviation. The PLS evaluation could assist in the determination of a correlation between the anti-radical activity (1/IC_50_) and phytochemical constituents of extracts. From the PLS biplot that combines score and loading plots, three clusters were clearly noted (Figure 4A,B), as previously shown by the PCA score plot.

From the PLS biplot of the FD samples extracted with the different ethanol ratios (Figure 4A), the 80% extract was clearly separated from the other extracts as previously shown by PCA. These samples were located adjacent to FRAP and antioxidant and α-glucosidase inhibitory activities, suggesting a strong correlation between them. In terms of drying methods, FD was obviously separated from the other dried extracts, situated near TPC, FRAP and antioxidant and α-glucosidase inhibitory activities (Figure 4B). The high levels of catechin, epicatechin, rutin, quercetin and chlorogenic, caffeic, malic and gallic acids in 80% ethanol and FD extracts might be the reason for the correlation. For the variability among ethanol ratios extracts, the polarity index (PI) of the solvent and the solubility of metabolites are the possible factors that might affect the bioactivity and the recovery of bioactive compounds. These metabolites were the greatest contributors to the bioactivities, considering their VIP values. The highest tested activity and phytochemical constituents explained the separation of the FD extracts from the others. Several researchers suggested that FD is the preferred drying method for retaining bioactive compounds [7,22]. Another study recommended that FD and AD are advantageous in preserving plant efficiency and bioactivity [13]. This agrees with other findings with respect to the role of phenolics in these biological activities. It has been reported by many researchers that chlorogenic acid [24], ellagic acid [25], quercetin and quercetin rhamnoside exhibit prominent antidiabetic properties [24,25,26,27,28]. In fact, quercetin is also used as a positive standard in these biological activities. This trend also supports the fact that the free radical-scavenging activity of a plant extract is mostly due to the phenolics content [29]. Oxygen free radicals increase during both type I and type II diabetes, and thus, antioxidants could have beneficial effects on the control of this disease [27]. An interesting finding is that hypophyllanthin was strongly correlated with DPPH and α-glucosidase inhibitory activities, showing a prominent contribution to these bioactivities with a high content in both FD and 80% ethanol extracts. This metabolite is a unique chemical marker of this plant, and it can be a potent antioxidant and α-glucosidase inhibitor. According to previous studies, the addition of low quantities of water to the ethanol or methanol augments its ability to extract bioactive compounds [29,30].

In the present study, the *Q*^2^ and *R*^2^ values were between 0.85 and 0.97. This suggested that all models met the criteria of the validation and prediction performances. The permutation tests with 100 permutations and cross validation showed that the PLS models were valid (Supplementary Figure S3). The correlation coefficients (*R*) were calculated to determine and verify the relationship between variables. The different variables for the established models were also validated from the root mean square error of cross-validation (*RMSECV*). For this validation, the root-mean square of errors of both estimation and prediction was similar, and these errors were reduced as the PC number included in the model increased (Supplementary Figure S4). These data also revealed that the PLS presented good models for the prediction and validation of the parameters. These results indicate that the *RMSECV* was low with excellent correlation coefficients (*R* > 0.9). This suggests that all models meet the criteria for great validation and prediction performances.

## 3. Materials and Methods 

### 3.1. Chemicals and Reagents

The chemicals and reagents that were used, including non-deuterated monopotassium phosphate (KH_2_PO_4_), deuterated methanol-*d*_4_ (CH_3_OH-*d*_4_), sodium deuterium oxide (NaOD), deuterium oxide (D_2_O), trimethylsilyl propionic acid-*d*_4_ sodium salt (TSP) and α-glucosidase enzyme, were supplied by Merck (Darmstadt, Germany). Liquid nitrogen was purchased from Linde Company (Petaling Jaya, Malaysia).

### 3.2. Plant Material and Drying Treatments

*Phyllanthus niruri* was planted at Sendayan Commodity Development Center in Negeri Sembilan, Malaysia. The plant was planted under the shade in a located plot. The temperature and relative humidity were suitable for good plant growth. Prior to planting, the soil was turned, treated, fertilized, and covered with a black plastic for better growth conditions. The irrigation was provided automatically. The organic fertilization and weed removal were also done weekly. However, the plant treatment using pesticide was avoided throughout the growth period. For collection, whole plants were harvested in the early morning on the same day, cleaned to remove dirt or sand and then pre-dried with tissue paper. The samples were subjected to drying via three methods, including air (AD), freeze (FD) and oven (OD) drying. For FD, each sample was immediately ground in a mortar and pestle under liquid nitrogen. The powdered sample was transferred to a plastic tube, stored overnight at −80 °C and freeze-dried to total dryness. For OD, each sample was dried in a Memmert Universal (Schwabach, Germany) laboratory oven at 40 °C for 48 h with maximum air velocity to avoid moisture increase. For AD, each sample was dried at ambient temperature over a period of six days. All of the samples were ground to a very fine powder in a laboratory blender, sieved to similar particle size (0.70 mm) and stored at 4 °C prior to analysis.

### 3.3. Extraction of Samples

Each dried sample (20 g) was immersed in 500 mL of ethanol at different ratios (0%, 50%, 70%, 80%, and 100%) in an amber bottle. The mixtures were shaken to mix the samples with the solvent and then sonicated for 1 h in a sonicator at a controlled temperature of 25 °C. Thereafter, the mixture was filtered through filter paper and cotton twice to completely remove any debris. The residual solvents were eliminated using a rotary evaporator under a partial vacuum at 40 °C. The samples were frozen in a deep freezer at −80 °C and then lyophilized in a freeze drier to ensure removal of the residual water. The concentrated extracts were stored in an amber bottle in a refrigerator at 4 °C prior to analysis.

### 3.4. Amino Acid Content Determination

The amino acids content of the samples was determined according to the Waters™ PICO.TAG™ method (P/N 88131) [21]. An internal standard (α-aminobutyric acid) was prepared by dissolving 0.2578 g in 0.1 M HCl and adjusting the volume to 1 L. Mobile phase A contained 0.1 M ammonium acetate at pH 6.5, and mobile phase B had 0.1 M ammonium acetate with acetonitrile and methanol (44:46:10) at pH 6.5. The pH was adjusted using acetic acid. The re-drying solution was prepared by mixing methanol, water and triethylamine at a ratio of 2:2:1, and the derivation reagent contained phenylisothiocyanate (PITC), deionized water, trimethylamine and methanol at a ratio of 1:1:1:7.

For analysis, a 4/x g of sample was weighed, where x represents the percentage of protein in the respective samples, which was determined earlier. The samples were placed in test tubes with covers, and 15 mL of 6 M HCl was added to the sample and vortexed. The test tubes were put in an oven at 110 °C for 24 h. After cooling, 10 mL of internal standard was added to the test tubes. The hydrolyzed samples were poured into 50-mL volumetric flasks, brought to volume with deionized water, and then filtered through filter paper (0.2 μm cellulose membrane filter). A 10 μL aliquot of the hydrolyzed sample was placed into a Durham tube. The same amount of mixed amino acids standard was also inserted into another Durham tube. The tubes were dried under vacuum for 30 min, and 20 μL of re-drying solution was added to each tube before the mixtures were vortexed. The tubes were vacuum dried for another 30 min, mixed with 20 μL of derivatization reagent and vortexed. The tubes were left at room temperature for 20 min and were vacuum dried for 30 min until dry. High-performance liquid chromatography (HPLC) with an RP 18 column (3.9 mm × 15 cm) was used to identify and quantify the amino acids. Before injection into the HPLC, the samples and standards were mixed with 100 μL of mobile phase A and vortexed for 15 min. The injection volumes of the standard, blank and samples were 8, 8 and 20 μL, respectively. 

### 3.5. Nuclear Magnetic Resonance Measurement

The ^1^H-NMR and two-dimensional (2D) NMR determinations were implemented using a 500 MHz Varian INOVA NMR spectrometer (Varian Inc., Palo Alto, CA, USA), functioning at a frequency of 499.887 MHz at room temperature (25 °C). The 2D NMR included *J*-resolved and heteronuclear multiple bond coherence (HMBC). The extraction procedures were carried out as previously reported [13]. A 10 mg sample was placed in a 2 mL Eppendorf tube, and a total of 0.75 mL of a 1:1 mixture of CH_3_OH-*d*_4_ and KH_2_PO_4_ buffer (pH 6.0) in D_2_O containing 0.1% TSP was added. The mixture was vortexed for 1 min and ultrasonicated for 15 min at room temperature. Then, the mixture was centrifuged at 13,000 rpm for 10 min to obtain a clear supernatant, and 0.6 mL of the supernatant was transferred to an NMR tube to perform NMR analysis.

The NMR tubes were labeled and immediately subjected to ^1^H-NMR measurements using a preset setting for all samples. The required time for each ^1^H-NMR spectrum was 3.53 min, which contains 64 scans with a width of 20 ppm. The time involved for *J*-resolved spectrum was 50 min and 18 s containing 8 scans per 128 increments for the axis of spin-spin coupling constant with spectral widths of 66 Hz and 8 K for the chemical shift axis with spectral widths of 5000 Hz. The relaxation delay was 1.5 s. The HMBC spectra were obtained by 64 scans, giving an achievement time of 6 h, 9 min and 9 s. Chenomx software (v. 6.2, Edmonton, AB, Canada) was applied for the phasing and baseline corrections with a reliable setting for all sample spectra. By applying the previously described method, ^1^H-NMR was run as previously reported and with some known metabolites for comparison.

### 3.6. Bucketing of ^1^H Nuclear Magnetic Resonance Spectra and Multivariate Data Analysis

Chenomx software (v. 6.2, Edmonton, AB, Canada) was implemented to perform the bucketing of ^1^H-NMR spectra. All spectra were automatically binned to ASCII files with the same parameters, which included a spectral width (δ 0.04) forming a region of 0.5–10.0 ppm to obtain a total of 230 integrated areas per NMR spectrum. The chemical shift ranges of δ 4.70–5.00 and δ 3.27–3.35, which represent water and residual methanol, respectively, were excluded.

### 3.7. Statistical Analysis

After binning the NMR spectra, multivariate data analysis (MVDA) by principal component analysis (PCA) and partial least square (PLS) were performed with SIMCA-P software (v. 13.0, Umetrics, Umeå, Sweden) using the Pareto scaling method. In the obtained data matrix with *N* = 90 rows (observations) and *K* = 243 (variables), the NMR chemical shift was the *X* variable, and the sample names were the observations. The bioassay results were expressed as the mean of six values with standard deviation. Analysis of variance (ANOVA) was applied to evaluate the significant difference between the obtained bioassay data at a confidence interval of 95%. All analyses were carried out using the InStat V2.02 statistical package (GraphPad Software, San Diego, CA, USA). The IC_50_ values for both DPPH and α-glucosidase were converted into anti-radical activity and defined as 1/IC_50_ because this parameter has the same trend as the activity.

## 4. Conclusions

The aim of this work was to use a ^1^H-NMR-based metabolomics approach to evaluate the effects of drying methods on the nutritional and metabolite compositions and bioactivity of *P. niruri*. As a function of different drying methods and various ethanol ratios, there was remarkable variance in the metabolomes. The FD material extracted with 80% ethanol was found to exhibit the highest antioxidant and α-glucosidase inhibitory activities. The PLS results revealed that the identified phenolics and hypophyllanthin were strongly correlated with the antioxidant and α-glucosidase inhibitory activities, suggesting their great contributions to these activities. The ^1^H-NMR coupled with MVDA was a useful tool for discriminating three dried *P. niruri* samples extracted with several ethanol ratios. The NMR also provides an instantaneous assignment of a wide range of metabolites, offering the simultaneous metabolome profiling of *P. niruri* extracts. NMR was successfully applied to optimize the postharvest conditions, including the drying and extraction parameters. High levels of *P. niruri* extracts can be incorporated into other food products to help in protecting human bodies against degenerative diseases. These findings show that *P. niruri* has potential as a beneficial source of phytochemicals and may be exploited further as a functional food product. 

## Figures and Tables

**Table 1:** Amino acid content of the three dried *Phyllanthus niruri* extracts.

Amino Acids	Freeze Drying (FD)	Oven Drying (OD)	Air Drying (AD)
Aspartic acid	3.18 ^a^ ± 0.13	1.85 ^b^ ± 0.19	2.10 ^c^ ± 0.11
Glutamic acid	9.33 ^a^ ± 0.18	4.32 ^b^ ± 0.03	4.59 ^b^ ± 0.32
Serine	2.33 ^a^ ± 0.14	0.98 ^b^ ± 0.09	0.95 ^b^ ± 0.14
Glycine	2.10 ^a^ ± 0.16	0.91 ^b^ ± 0.06	0.97 ^b^ ± 0.09
Histidine	1.24 ^a^ ± 0.10	0.56 ^b^ ± 0.18	0.42 ^c^ ± 0.02
Arginine	2.48 ^a^ ± 0.03	1.48 ^b^ ± 0.33	0.69 ^c^ ± 0.08
Threonine	1.55 ^a^ ± 0.03	0.61 ^b^ ± 0.24	0.71 ^b^ ± 0.02
Alanine	2.48 ^a^ ± 0.11	1.50 ^b^ ± 0.19	1.46 ^b^ ± 0.30
Proline	2.05 ^a^ ± 0.06	1.20 ^b^ ± 0.04	2.88 ^c^ ± 0.05
Tyrosine	1.00 ^a^ ± 0.10	0.24 ^b^ ± 0.03	0.34 ^b^ ± 0.05
Valine	2.60 ^a^ ± 0.10	1.15 ^b^ ± 0.03	1.08 ^b^ ± 0.09
Methionine	0.34 ^a^ ± 0.06	0.19 ^b^ ± 0.04	0.11 ^b^ ± 0.01
Cystine	9.50 ^a^ ± 0.54	4.17 ^b^ ± 0.22	4.79 ^b^ ± 0.52
Isoleucine	2.08 ^a^ ± 0.04	0.87 ^b^ ± 0.02	0.82 ^b^ ± 0.08
Leucine	3.49 ^a^ ± 0.08	1.43 ^b^ ± 0.02	1.34 ^b^ ± 0.08
Phenylalanine	4.01 ^a^ ± 0.42	2.89 ^b^ ± 0.06	2.84 ^b^ ± 0.16
Lysine	14.10 ^a^ ± 0.32	5.76 ^b^ ± 0.06	6.42 ^b^ ± 0.58

Represented values are the means ± standard deviations; Different superscript letters for the same amino acid comparing the three drying methods showed significant (*p* < 0.05) difference.

**Table 2:** Nuclear magnetic resonance (NMR) characteristic signals of identified metabolites in *Phyllanthus niruri* extracts.

Metabolites	^1^H-NMR Characteristic Signals
**Primary metabolites:**	
β glucose	δ 4.59 (d, *J* = 8.0 Hz)
α glucose	δ 5.19 (d, *J* = 4.0 Hz)
Sucrose	δ 5.41 (d, *J* = 3.5 Hz)
Fructose	δ 4.18 (d, *J* = 8.5 Hz)
Fatty acid	δ 1.33–1.25 (m)
Formic acid	δ 8.47 (s)
Acetic acid	δ 1.93 (s)
Choline	δ 3.21 (s)
Alanine	δ 1.49 (d, *J* = 7.5 Hz), δ 3.72 (q)
d-l-Valine	δ 1.07 (d, *J* = 7.0 Hz), δ 1.02 (d, *J* = 7.0 Hz)
Leucine or isoleucine	δ 0.98 (d, *J* = 7.5 Hz)
**Secondary metabolites:**	
Quercetin 3-*O*-glucoside	δ 6.45 (d, *J* = 2.0 Hz), δ 7.37 (d, *J* = 2.0 Hz), δ 7.01 (d, *J* = 8.0 Hz), δ 7.33 (dd, *J* = 8.5, 2.0 Hz), δ 5.16 (d, *J* = 8.0 Hz)
Catechin	δ 6.45 (d, *J* = 2.0 Hz), δ 7.37 (d, *J* = 2.0 Hz), δ 7.01 (d, *J* = 8.0 Hz), δ 7.33 (dd, *J* = 8.5, 2.0 Hz), δ 5.40 (d, *J* = 7.6 Hz), δ 2.56 (dd, *J* = 7.5, 16.0 Hz), δ 2.84 (m)
Quercetin 3-*O*-α-rhamnoside	δ 6.45 (d, *J* = 2.0 Hz), δ 7.37 (d, *J* = 2.0 Hz), δ 7.01 (d, *J* = 8.0 Hz), δ 7.33 (dd, *J* = 8.5, 2.0 Hz), δ 5.48 (d, *J* = 1.0 Hz). Methyl signal: δ 0.91 (d, *J* = 1.1 Hz)
Epicatechin	δ 6.45 (d, *J* = 2.0 Hz), δ 7.37 (d, *J* = 2.0 Hz), δ 7.01 (d, *J* = 8.0 Hz), δ 7.33 (dd, *J* = 8.5, 2.0 Hz), δ 4.97 (d, *J* = 8.0 Hz), δ 7.05 (s), δ 6.95 (s)
Rutin	δ 6.51 (d, *J* = 2.0 Hz), δ 7.59 (dd, *J* = 8.5, 2.0 Hz), δ 7.67 (d, *J* = 2.0 Hz). Anomeric protons (glucosyl δ 4.97 (d, *J* = 8.0 Hz), rhamnosyl δ 4.54 (d, *J* = 1.0 Hz)
Chlorogenic acid (5-*O*-caffeoyl quinic acid)	δ 7.20 (s), signal for caffeoyl δ 7.62 (d, *J* = 16.0 Hz), δ 6.37 (d, *J* = 16.0 Hz) and quinic δ 4.09 (m), δ 2.63 (dd, *J* = 1.3, 5.0 Hz), δ 1.9 (d, *J* = 10.0 Hz), δ 7.20 (s)
Gallic acid	δ 7.07 (s), 7.15 (s)
Ellagic acid	δ 7.46 (s), 7.69 (s)
Malic acid	δ 2.72 (d, *J* = 16.0 Hz), δ 6.37 (d, *J* = 16.0 Hz)
Quinic acid	δ 4.09 (m), δ 2.63 (dd, *J* = 1.3, 5.0 Hz), δ 4.23 (d, *J* = 7.0 Hz)
Caffeic acid	δ 7.13 (d, *J* = 2.0 Hz), δ 6.87 (d, *J* = 9.0 Hz)
Phyllanthin	δ 2.05 (m), δ 2.70 (d, *J* = 8.0 Hz), δ 3.29 (d, *J* = 9.0 Hz), δ 3.33 (s), δ 3.81 (s), δ 6.61 (dd, *J* = 8.5, 2.0 Hz), δ 6.82 (d, *J* = 8.0 Hz)
Hypophyllanthin	δ 1.93 (m), δ 2.79 (d, *J* = 8.0 Hz), δ 3.29 (s), δ 3.31 (s), δ 3.85 (s), δ 4.08 (d, *J* = 7.2 Hz), δ 5.61 (d, *J* = 2.0 Hz), δ 5.72 (d, *J* = 2.0 Hz), δ 6.29 (s), δ 6.65 (d, *J* = 2.0 Hz), δ 6.71 (d, *J* = 5.0 Hz)

Run with deuterated methanol-*d*_4_ (CH_3_OH-*d*_4_) and deuterium oxide (D_2_O) buffer.

## Supplementary Materials

Supplementary materials are available online.
