# Supplementary material for: Characterization of Metabolite Profile in Phyllanthus niruri and Correlation with Bioactivity Elucidated by Nuclear Magnetic Resonance Based Metabolomics
Source: Molecules. 2017 May 30;22(6):902. doi: 10.3390/molecules22060902 (PMC6152626; doi:10.3390/molecules22060902)
Supplement: Supplementary file 1 [file molecules-22-00902-s001.pdf]

Supplemental Material

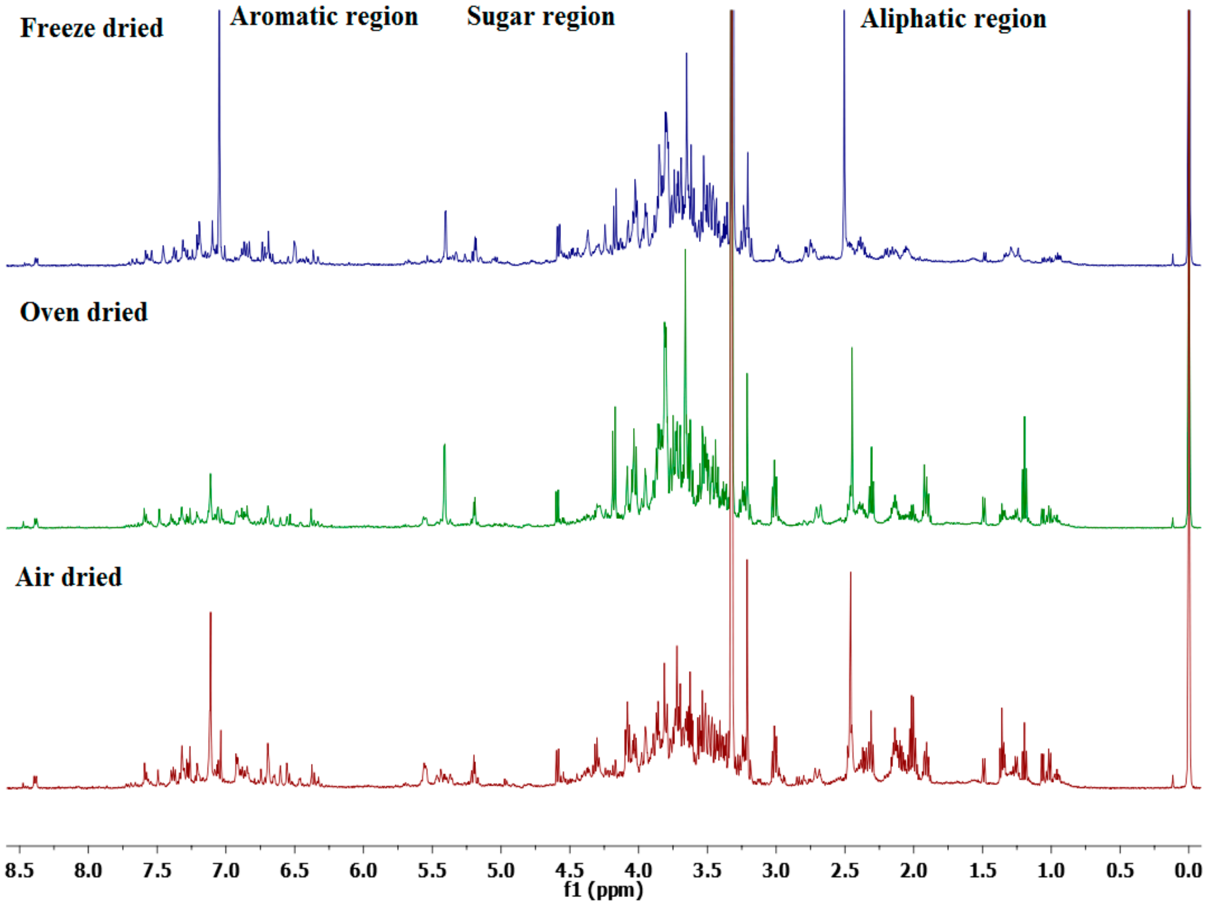

Figure S1. Overlay ^{1}H NMR spectra of the three dried *P. niruri* extracts

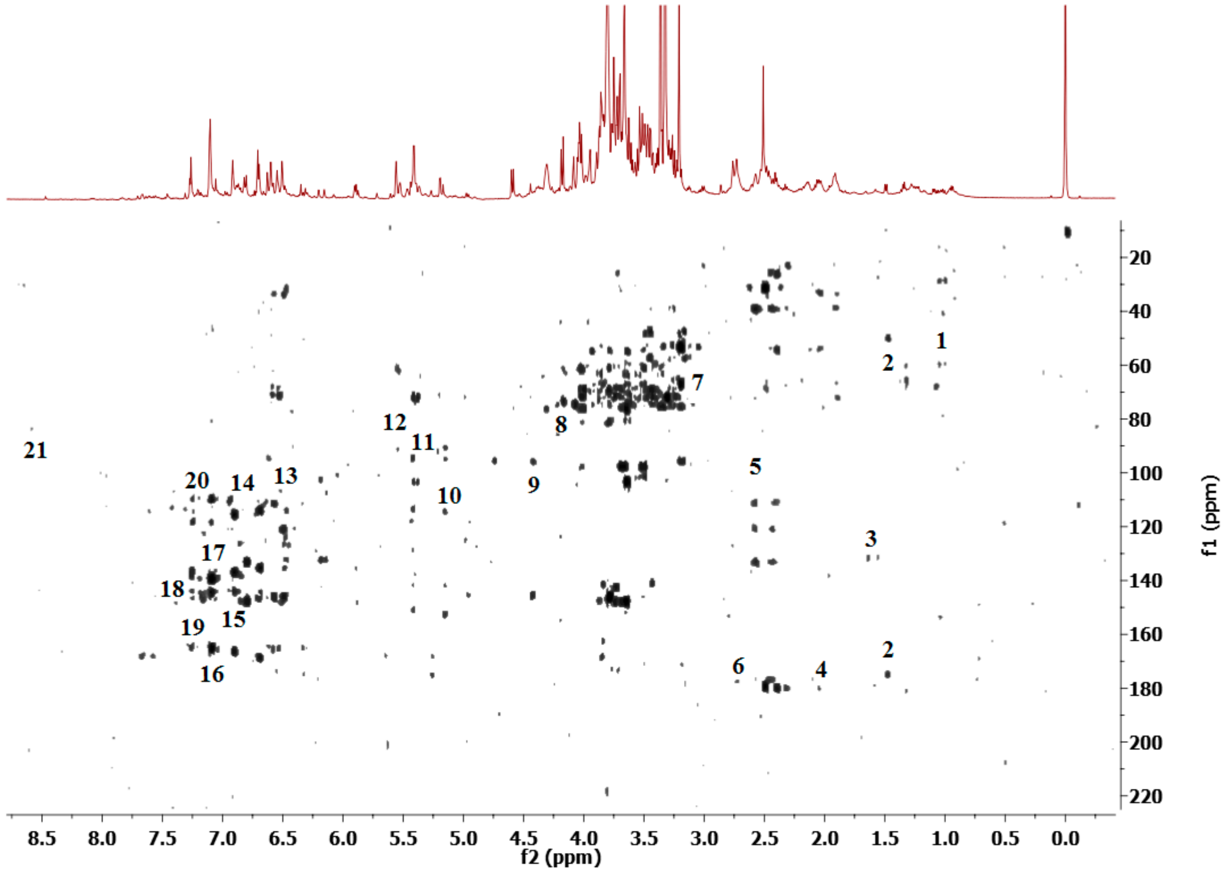

Figure S2. Representative HMBC spectra of *P. niruri* extract

Assignments: 1, Leucine; 2, alanine; 3, valine; 4, acetic acid; 5, catechin; 6, malic acid; 7, choline; 8, fructose; 9, β-glucose; 10, α-glucose; 11, sucrose; 12, hypophyllanthin; 13, rutin; leucine; 14, phyllanthin; 15, epicatechin; 16, caffeic acid; 17, quercetin derivatives; 18, ellagic acid; 19, gallic acid; 20, chlorogenic acid; and 21, formic acid

DPPH

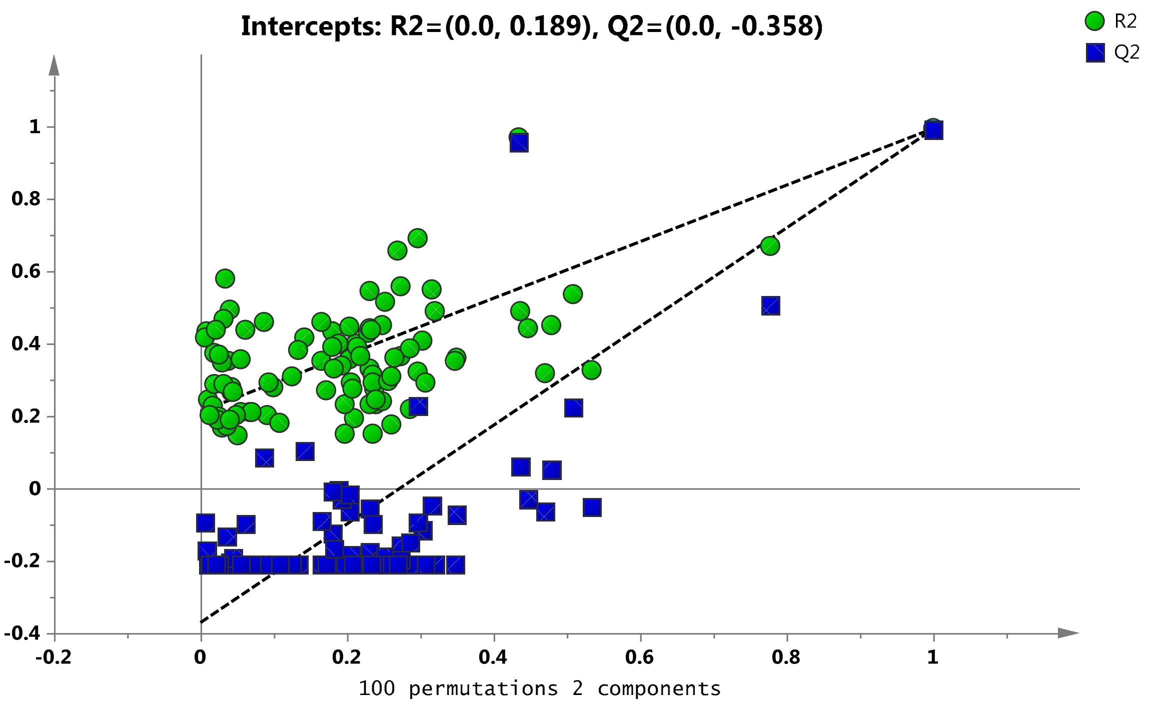
Intercepts: R2=(0.0, 0.189), Q2=(0.0, -0.358)
R2
Q2
1
0.8
0.6
0.4
0.2
0
-0.2
-0.4
-0.2
0
0.2
0.4
0.6
0.8
1
100 permutations 2 components

FRAP

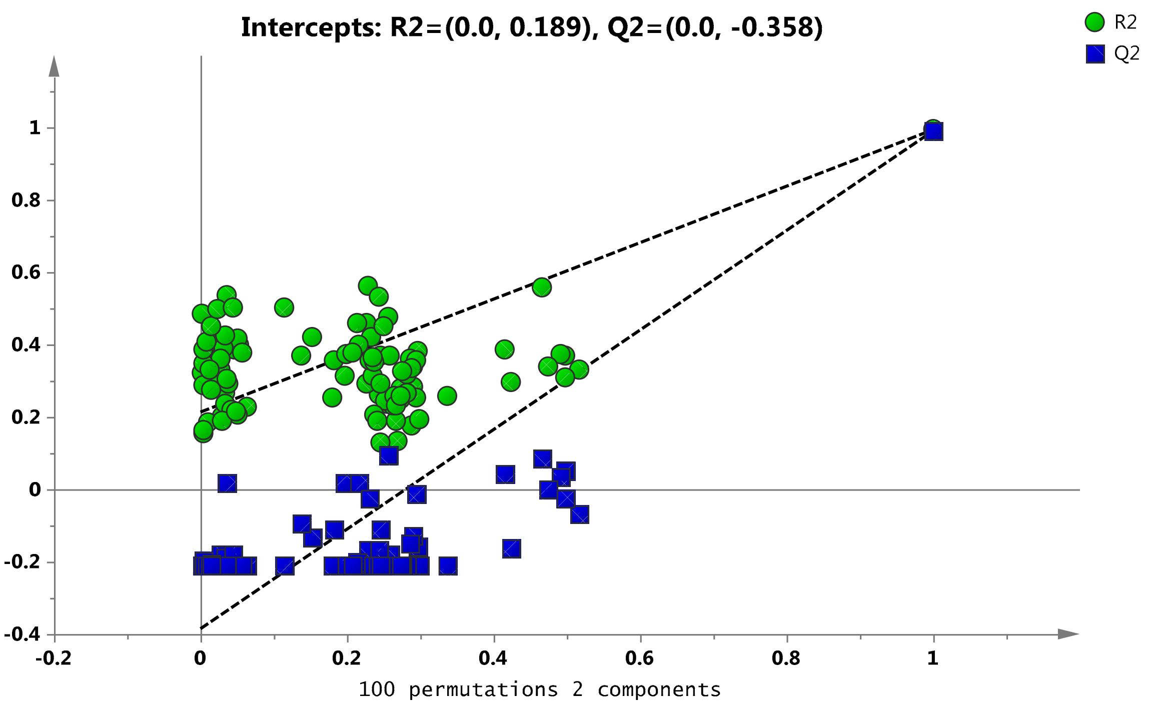
Intercepts: R2=(0.0, 0.189), Q2=(0.0, -0.358)
R2
Q2
1
0.8
0.6
0.4
0.2
0
-0.2
-0.4
-0.2
0
0.2
0.4
0.6
0.8
1
100 permutations 2 components

Alpha glucosidase

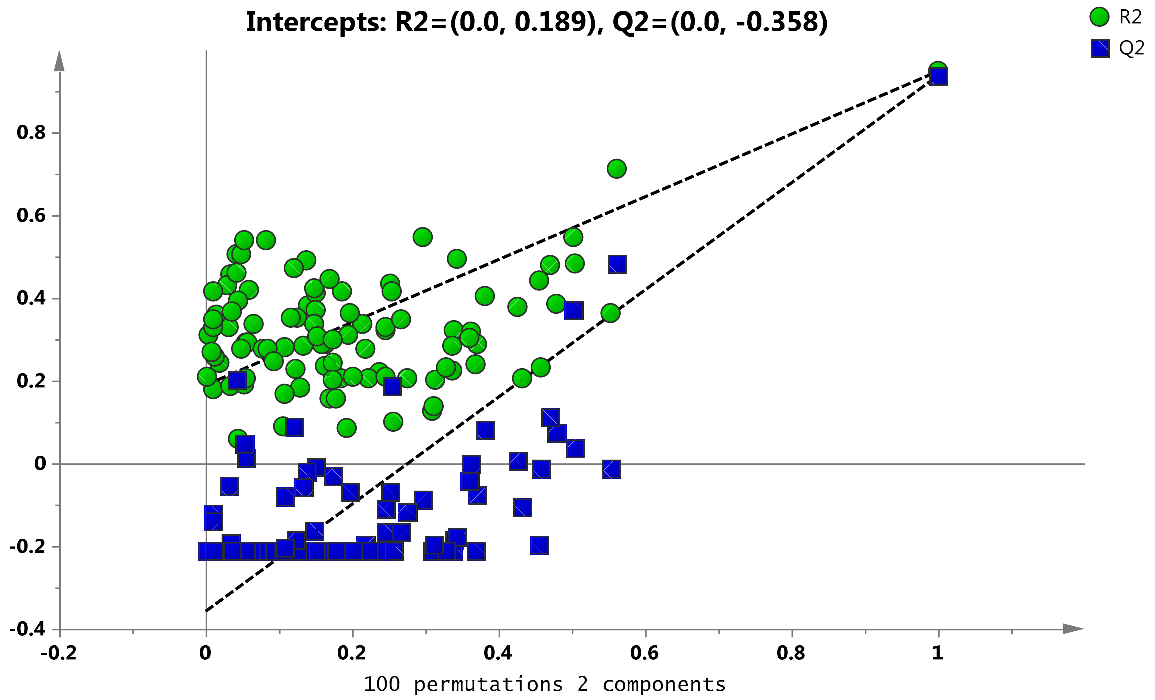

Figure S3. The permutations for validation of partial least square (PLS) model

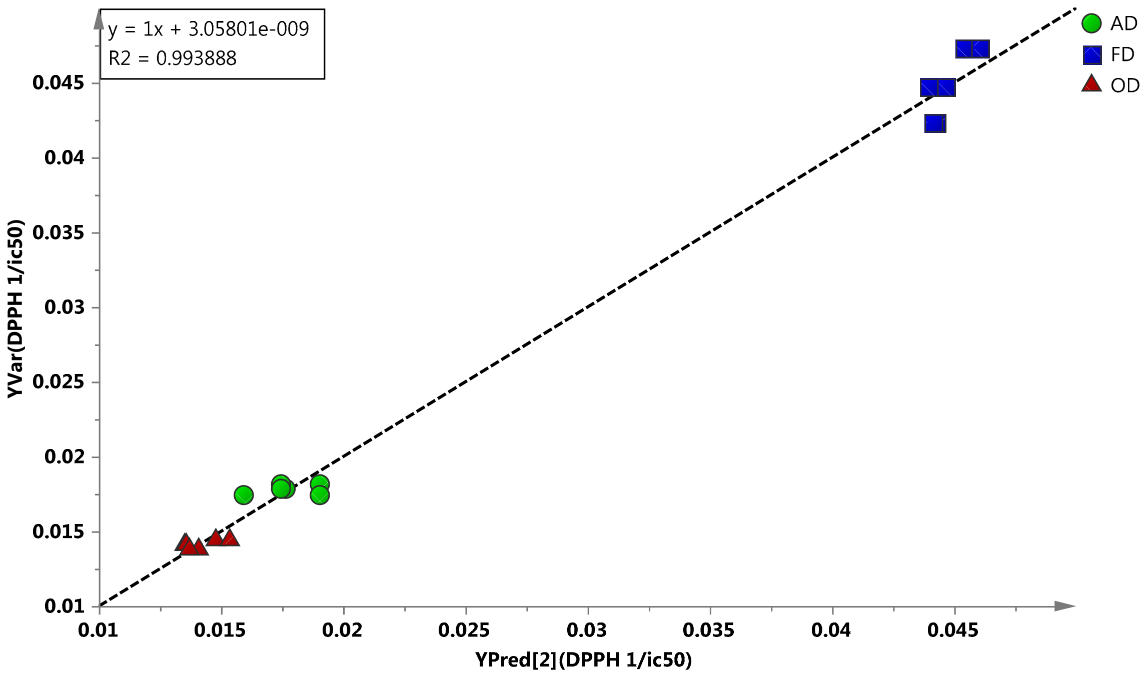

RMSEE = 0.00117505   RMSEcv = 0.00147757

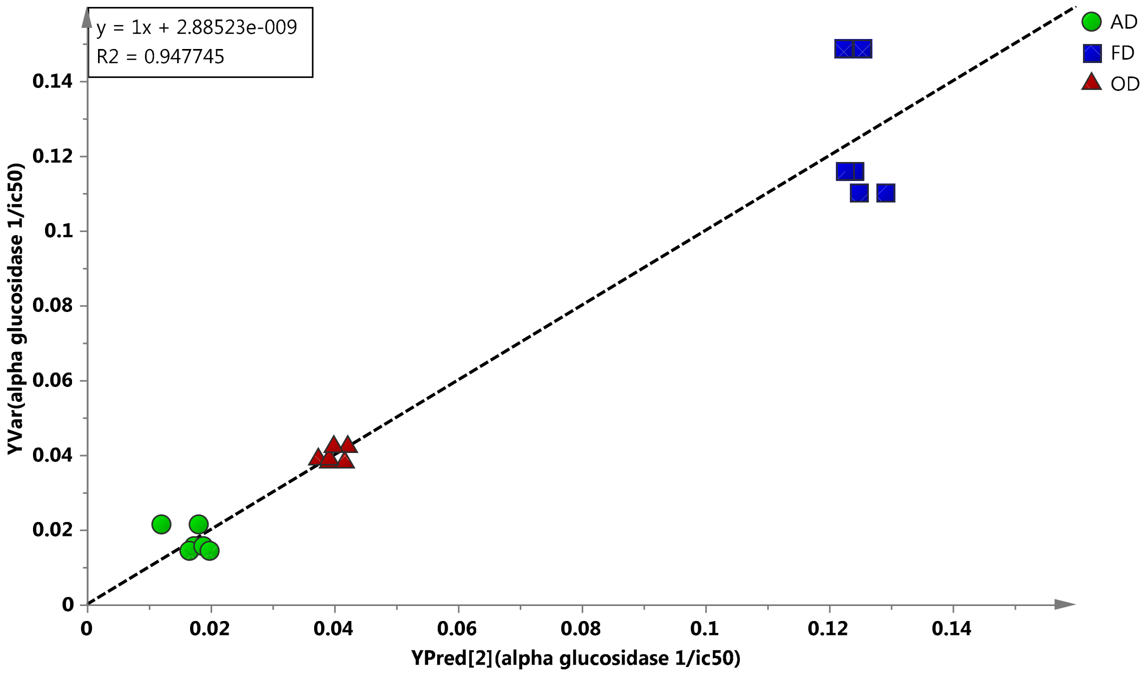

RMSEE = 0.01191   RMSEcv = 0.0122639

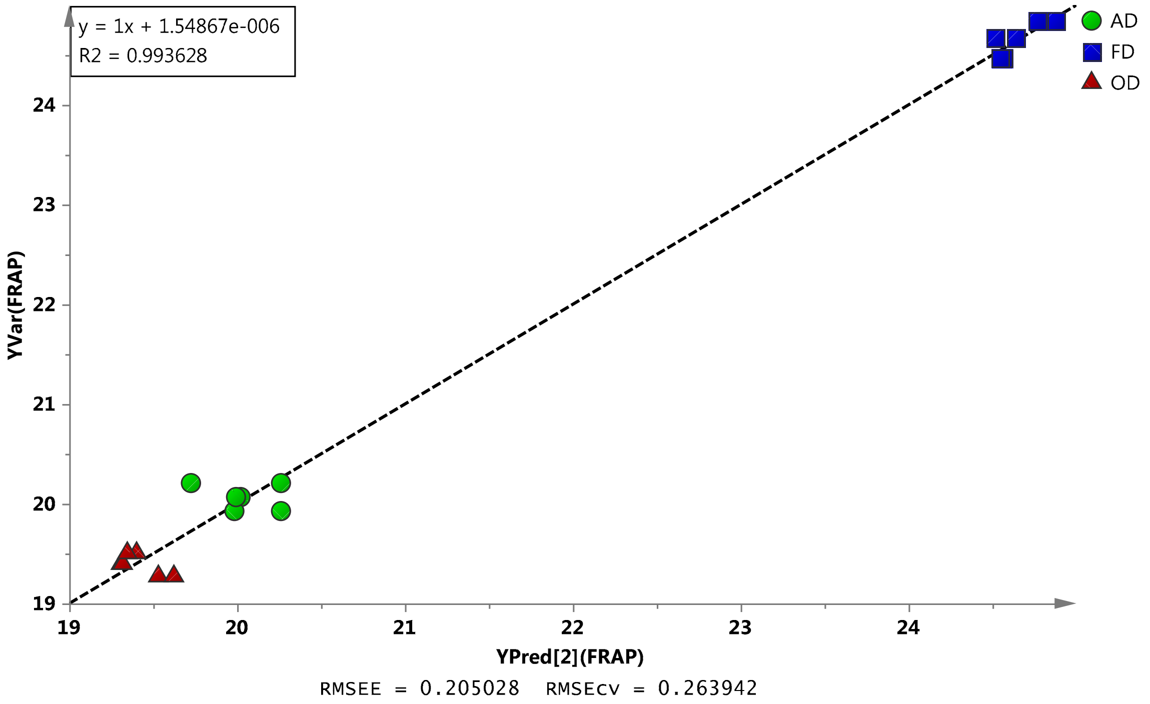

AD – Air drying; OD – Oven drying; FD – Freeze drying

Figure S4. Observed versus predicted plots of partial least square (PLS) model
